# WaaS architecture-driven depressive mood status quantitative analysis based on forehead EEG and self-rating tool

**DOI:** 10.1186/s40708-018-0093-y

**Published:** 2018-12-05

**Authors:** Zhijiang Wan, Hao Zhang, Jianhui Chen, Haiyan Zhou, Jie Yang, Ning Zhong

**Affiliations:** 1 Department of Life Science and Informatics, Maebashi Institute of Technology, Maebashi, 3710864 Japan; 2 College of Economics and Management, Nanjing Forestry University, Nanjing, 210037 China; 3 College of Electronic Information and Control Engineering, Beijing University of Technology, Beijing, 100124 China; 4 International WIC Institute, Beijing University of Technology, Beijing, 100088 China; 5 Beijing Anding Hospital of Capital Medical University, Beijing, 100088 China

**Keywords:** WaaS, Forehead EEG, Q-Log, Depression quantitative analysis, Ontology technology, Regression analysis

## Abstract

**Background:**

Although the objective depression evaluation is a hot topic in recent years, less is known concerning developing a pervasive and objective approach for quantitatively evaluating depression. Driven by the Wisdom as a Service architecture, a quantitative analysis method for rating depressive mood status based on forehead electroencephalograph (EEG) and an electronic diary log application named quantitative log for mental state (Q-Log) is proposed. A regression method based on random forest algorithm is adopted to train the quantitative model, where independent variables are forehead EEG features and the dependent variables are the first principal component (FPC) values of the Q-Log.

**Results:**

The Leave-One-Participant-Out Cross-Validation is adopted to estimate the performance of the quantitative model, and the result shows that the model outcomes have a moderate uphill relationship (the average coefficient equals 0.6556 and the *P* value less than 0.01) with the FPC values of the Q-Log. Furthermore, an exemplary application of knowledge sharing, which is developed by using ontology technology and Jena inference subsystem, is given to illustrate the preliminary work for annotating data and facilitating clinical users to understand the meaning of the quantitative analysis results.

**Conclusions:**

This method combining physiological sensor data with psychological self-rating data could provide new insights into the pervasive and objective depression evaluation processes in daily life.

## Introduction

Neuropsychiatric diseases are the worlds leader in years lived with disability, accounting for almost 30% of total years lived with disability [1, 2]. Among the various neuropsychiatric diseases, the major depressive disorder (MDD) is widely distributed in the worldwide populations and it is one of the leading causes of disability in both adolescents and adults [3–5]. It is a mental illness that involves several symptoms such as low mood, feeling worthless or guilty, loss of interest in activities, weight loss or gain, insomnia, anxiety, being tired, trouble concentrating, thoughts of suicide.

Traditional diagnostic and evaluation approaches for MDD are based on patient interviews, which attempt to give a comprehensive evaluation for several depressive symptoms and form a treatment decision. However, such kind of depression evaluation method provides a subjective assessment for the depressive symptoms and frequently shared with other maladies [6–9]. That is, reliance upon clinical assessments and patient interviews for diagnosing MDD is frequently associated with misdiagnosis and suboptimal treatment outcomes. In addition, the most clinical tools, such as Hamilton Depression Scale (HAMD) and Beck Depression Inventory (BDI), always have good test–retest reliability, which could reliably replicate the result more than once in the same situation and population [10]. Such tools are always used to provide a cross-sectional assessment of psychological status but unable to assess the daily change of psychological status. A continuous assessment on mood status with fine granularity might give circumstantial evidence to reflect the depression idiosyncrasy of patients [10]. Moreover, fear of discrimination is one of the difficulties for depression treatment [11]. Depressed people feel the world is deeply suspicious of them and is unlikely to befriend them. Researchers at Kings College London Institute of Psychiatry used detailed questionnaires to ask 1082 people being treated for depression in 35 different countries about their experiences of discrimination. The study shows that around 71% actively wish to conceal their depression from other people, which has led to concerns that people with depression might not seek help, and doing so would more than likely make their condition become chronic.

Objectively quantifying the severity of depression symptoms might be a viable way to decline the misdiagnosis rate and improve the effectiveness of treatment outcomes. Compared with clinical scales, objective methods based on physiological data not only have the advantage of consistency result for quantitatively assessing depression, but also provide a continuous assessment in a daily granularity. More importantly, using wearable devices to detect the physiological data, those methods are easy to be implemented in the pervasive environment and avoid the privacy disclosure. Therefore, there is an increasing interest in studying objective methods for quantitatively evaluating depression based on physiological and behavioral data [10, 12–21]. In this study, we mainly focus on finding out a feasible and pervasive way to objectively quantify depression symptoms based on physiological data. To gain this, three questions should be dealt with:

1. Which physiological data is eligible to provide a quantitative evaluation for which symptom?
2. How to validate the effectiveness of the quantitative evaluation method?
3. How to make the method pervasively and apply it in clinical field of depression?

For the first question, depression is defined as a psychiatric illness that at least one of the depressive symptoms must be depressed mood or loss of interest in activities, and the symptom should be continuously experienced for at least 2 weeks. Additionally, clinical rating scales for MDD are all contain items for rating depressed mood, such as feeling guilty, anxiety and other emotional status. Therefore, the depressed mood is a critical symptom for diagnosing and evaluating depression. The studies showed that faulty mood regulation by brain is one of the causes of depression [22]. Based on the various physiology measurement tools, such as functional magnetic resonance imaging (fMRI), electroencephalogram (EEG), gene, many studies tried to measure the psychological data and measure the brain disorder. As one of the measurement tools, quantitative measurement of brain electrical signals taken from the EEG is a neuroimaging technique with clear practical advantages as it does not involve invasive procedures, is widely available, easy to administer, well tolerated and has a relatively low cost. The pervasive and persistent nature of depressive symptoms has made scalp-recorded EEG as an appropriate approach for understanding the causes of major depressive disorder. Techniques based on EEG are used to investigate the predictive utility with regard to mood improvement. Many studies show that anterior regions of the brain may modulate the differential effects of emotional arousal on the information-processing capacities of the cerebral hemispheres. Deldin et al. designed a cognitive experiment with four EEG recording blocks to find out the difference of EEG activities between controls and depressives; the study discovered that depressed responders further exhibit a cortical asymmetry of greater right relative to left activity in frontal areas [23, 24]. Kan et al. aimed at determining the differences of alpha waves between normal and depressive groups [24]. The conclusions show that the frontal lobe has much lower alpha waves in the depression group compared to the normal group. Many other studies are also show a high correlation between depressed mood and EEG wave in frontal areas of brain [25–27]. Therefore, it is a feasible way to objectively quantify depressive mood status based on forehead EEG data.

For the second question, current clinical methods for depression diagnosis and evaluation are still based on subjective clinical interview or clinician-rated scales. Few studies claimed to develop a quantitative method for evaluating depression to supersede traditional approaches; the majority prefer to choose clinical assessment tool as a ground truth to test the effectiveness of the quantitative evaluation method. Cummins et al. suggested that researchers should select a commonly used scale with clinical validity, i.e., HAMD, to quantify depression severity and even suicide risk [28]. They also claim that self-rated scale is a good choice, which is potentially more sensitive to detect changes than clinician-rated scale, to rate the severity in milder forms of depression. Sung et al. used noninvasive physiological and behavioral measures and visual analog self-rating scale to collect a novel dataset of physiology, behavior and emotional data over long-term periods [29]. They show that it is possible to create an individualized objective metric for depression based on simple physiological measures and have evidence that there are universal physiological features that can potentially be used to create a universal objective metric for depression. A desire quantitative evaluation method for evaluating depression severity is that the effectiveness is congruent with the traditional evaluation approaches used in the clinical treatment. That is, compared with the depression severity evaluation result rated by clinician-rated or self-rated tools, the effectiveness of the quantitative evaluation method could be validated.

For the third question, physiological data-based quantitative analysis is a promising way in adjunctive depression treatment. Quantitative EEG (QEEG) results offer a better picture of treatment effects with antidepressant medications than a medication algorithm according to a study presented by US Psychiatric and Mental Health Congress 2009 [30]. The study led by Bares et al. indicates that prefrontal QEEG cordance is a promising tool not only for predicting the response to certain antidepressants but also for repetitive transcranial magnetic stimulation (rTMS) treatment, with comparable predictive efficacy for both therapeutic interventions [31]. Griffeth et al. conclude that a quantitative methodology based on dual-echo measurement of blood flow and blood-oxygen-level-dependent (BOLD) responses is a promising tool for applying fMRI to disease and drug studies [32]. However, there exist some challenges in transferring the quantitative evaluation method for depression from clinical application to real world: (1) physiological measurement tools in hospital for depression treatment are cumbersome and difficult to be operated, which indicates that the tools are not suitable for making pervasive application; (2) physiological signal-based quantitative analysis application for depression is an interdisciplinary application, which involves clinical knowledge, data science and computer-aided engineering. It is difficult for the clinicians or the people who are not adept at using machine learning technology to understand the meaning of quantitative results; (3) quantitative analysis method is just a reference tool and insufficient to make a comprehensive treatment decision. That is, clinicians should consider conjointly analyzing the quantitative results associated with the contextual and clinical therapy information for making treatment decision.

In order to address the three questions mentioned above, the main contributions of this study are illustrated as follows:

1. A pervasive analysis method, which technical route follows the WaaS (Wisdom as a Service) architecture [33, 34], for objectively rating the depressive mood status using portable forehead EEG and self-rating tool is proposed. The WaaS architecture is a multidisciplinary, interdisciplinary research field for open intelligence service architectures and focuses on the Data–Information–Knowledge–Wisdom (DIKW) organization and transformation. The advantage of WaaS architecture is that it combines the data analysis results with experience knowledge to support the decision in the real world. In our case, we would like to integrate the quantitative evaluation results for mood status based on EEG data with clinical knowledge to generate clinical feedback automatically for clinicians.
2. An electronic diary log application named quantitative log for mental state (Q-Log) is utilized to rate the mood status by the patients themselves. By using the Q-Log, we require the subjects to self-rate their current mental state just like writing a diary. The principal component analysis (PCA) is used to process the Q-Log data. For each patient, a curve called first principal component (FPC) is extracted to reflect the overall tendency and daily change of the major depressive mood status. Furthermore, a regression analysis method based on random forest (RF) is selected for modeling the relationship between a scalar-dependent variable (FPC values of the Q-Log) and explanatory variables (EEG features).
3. In order to facilitate clinicians or other users to retrieve and query contextual information of inpatients, ontology technology is used to integrate data and annotate the connotation of related concepts and properties. The quantitative model based on the RF algorithm is manually transformed into several rules in Jena format, and the Jena inference API is utilized to create an inference model for obtaining the quantitative results. Eventually, an exemplary application named quantitative analysis for depression mood status and knowledge sharing is actualized.

## Material and methods

### Participants

We recruit subjects who are inpatients and undergoing clinical trials from Beijing Anding Hospital. Hitherto, 9 inpatients are recruited to join the experiment which lasted for 2 weeks. Every patient, who is willing to participate in this project, must meet the following inclusion and exclusion criteria:

Inclusion criteria: (1) inpatient with age 18–60; (2) right-handed and willingness to give written informed consent; (3) meet the Diagnostic and Statistical Manual of Mental Disorders (DSM-IV) criteria for major depressive episode without psychotic symptoms; (4) the Hamilton Depression Rating Scale with 17-items (HAM-D17) score $\ge$ 20 upon initial evaluation; (5) meet Mini-international Neuropsychiatric interview (MINI) criteria for unidirectional depression; (6) education level above junior high school; (7) not accept any psychotropic drug therapy in 2 weeks before experiment.

Exclusion criteria: (1) severe physical illness and unable to complete the questionnaire; (2) severe suicide risk; (3) brain damage with organic disease such as epilepsy and having other brain diseases with random discharge phenomenon; (4) prior emission computed tomography (ECT) treatment within the last 3 months; (5) in accordance with other psychiatric diagnosis criteria; (6) alcohol or drug abuse within the last 1 year or psychoactive addiction in the past and at present.

### Data acquisition

#### Portable EEG data acquisition

The forehead EEG data collected from Fp1 and Fp2 lobe simultaneously using B3 band. The B3 band is a portable EEG device equipping with the NeuroSky EEG biosensor (512 Hz sampling frequency and 12-bit ADC precision). All subjects are asked to record their EEG data in the resting state. The subjects are sitting on a sofa and keeping with eyes closed for 5 mins while not to think anything purposefully in a dim illuminated and acoustical room. They are also asked to maintain a minimum arousal level without falling into sleep. The subjects are required to collect their forehead EEG data twice daily (one time in the morning and evening, respectively) and continuously last for around 2 weeks.

#### Q-Log data acquisition

The data acquisition time of the Q-Log is congruent with the portable EEG data for each subject. The Q-Log is a visual analog scale which is deployed in the Android platform and operated via a slider bar. The subjects are asked to daily self-rate their current mood state just like recording or expressing the emotions by writing a diary. Forty adjectives of emotions vocabulary are cited from the Short Form of the Profile of Mood Status (POMS-SF) in Chinese [35, 36] and employed to instruct the subjects for rating their current mood states. The adjectives could be concluded into 7 mood categories, including tension–anxiety, depression–dejection, anger–hostility, fatigue–inertia, confusion–bewilderment, vigor–activity and self-mood relevant. Every adjective is rated on 100-point scale, that is, the emotion instructed by the adjective could be rated by a certain value range from 1 to 100. Five levels (not at all, a little, moderately, quite a bit and extremely) are used to give a detailed subdivision for the 100 points, and the interval between each of the levels has 25 points.

### Data analysis

#### Technical route for objectively quantifying depressive mood status

**Fig. 1:**
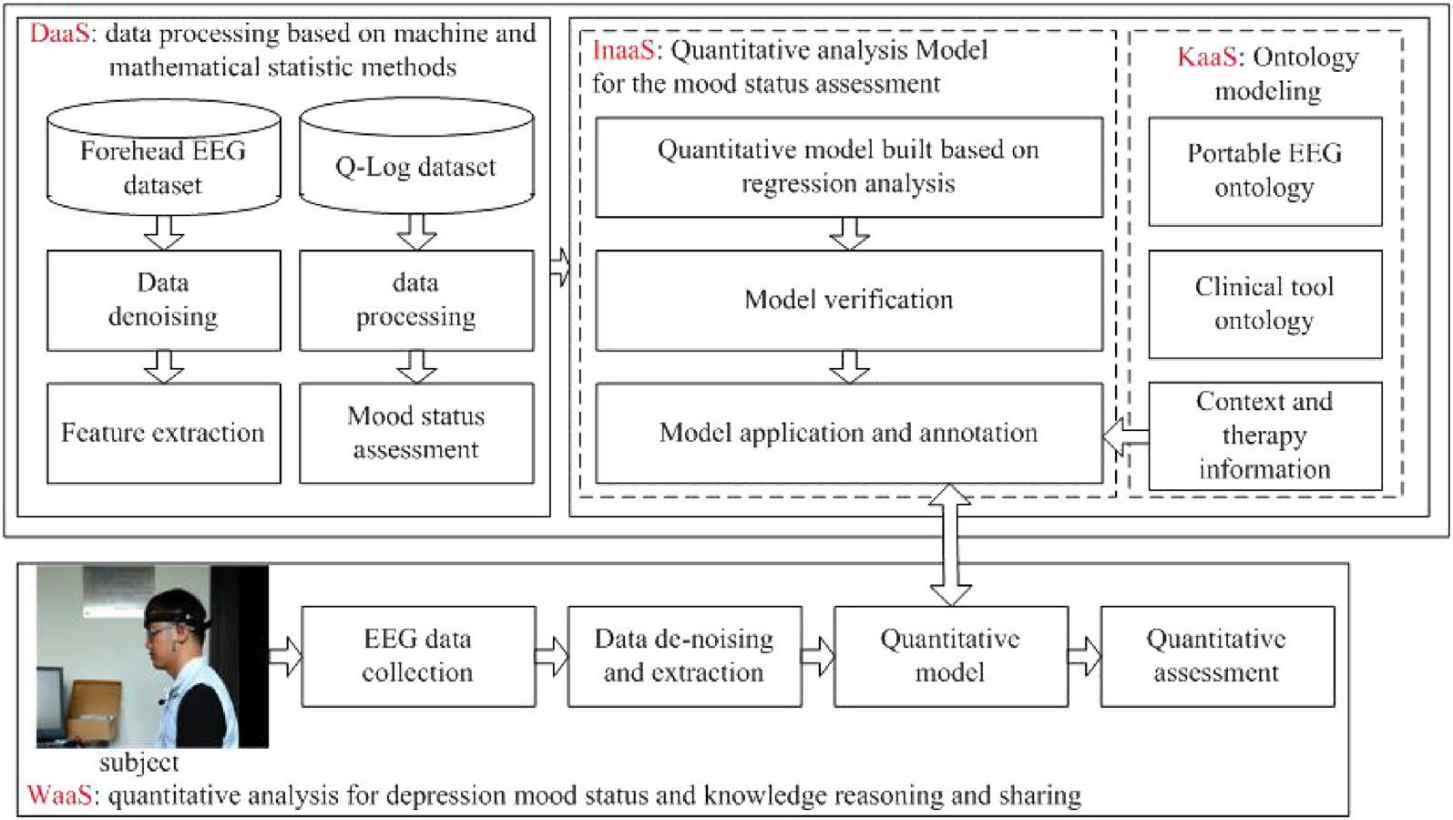
Technical route for quantitatively analyzing the depressive mood status based on forehead EEG and Q-Log data

Figure 1 shows the technical route for the method of quantitatively analyzing the depressive mood status. Following the WaaS architecture, the technical route can be illustrated as follows: in the DaaS layer, the EEG and Q-Log data of users are collected. After that, machine learning and mathematical statistics methods are used to process the collected data. In the KaaS layer, the personal context and clinical therapy information are structuralized via ontology technology. In order to facilitate implementing the conjoint analysis that associates with quantitative rating results, the collected data and the related data analysis method and results are also integrated into ontologies. In the InaaS layer, the regression analysis method is adopted to build quantitative model for rating depressive mood status. The Leave-One-Participant-Out Cross-Validation (LOPOCV) is utilized to verify the model effectiveness. Based on the Jena inference subsystem, the effective model is manually structuralized into several rules in Jena format, and a reasoning engine is constructed for quantitatively rating the major depressive mood status. In the WaaS layer, an exemplary application for quantitatively analyzing depressive mood status and knowledge sharing is provided.

#### Portable EEG data analysis

For each subject, every EEG sample is divided into several segments that overlapped by 50%. A data de-noising method based on discrete wavelet transform (DWT) is used to process the data. The DWT is used in two aspects: EEG data de-nosing and decomposition. For data de-nosing, a soft thresholding algorithm with ‘db5’ wavelet base is utilized to de-noise the raw EEG segment. For data decomposition, an eight-layer DWT with ‘db5’ wavelet base is used to decompose the de-noised EEG segment into five subband components. In this paper, 6 time series, which include the de-noised data and five subbands (delta, theta, alpha, beta and gamma), are extracted from the raw EEG segment. The frequency range of each subband is depicted as follows: delta (2–4 Hz), theta (4–8 Hz), alpha (8–16 Hz), beta (16–32 Hz) and gamma (32–64 Hz).

The linear, wavelet and nonlinear features are extracted from the 6 time series, respectively. The linear feature includes two categories: time and frequency domain feature. For the time domain, the features, such as mean, standard variation, prctile, kurtosis and skewness, are extracted. For the frequency domain, the Welch method with modified periodogram is applied to calculate power spectrum. In addition, the relative power, the absolute power and the relative power ratio of beta and theta subband are also calculated. The wavelet coefficients from third to seventh layer are extracted from the de-noised EEG segment and used as wavelet features. Based on the ratio of the energy of each layer, which can be represented by quadratic summations of the wavelet coefficients, a feature named wavelet entropy is calculated. For the nonlinear feature, the C0 complexity and the approximate entropy are extracted. Eventually, a feature vector with 176 dimensions (time domain: 54, frequency domain: 54, wavelet: 56 and nonlinear: 12) is extracted from the raw EEG segment.

#### Q-Log data analysis

The PCA is utilized to process the Q-Log data. The principal components of data matrix of each subject are calculated and represented by several eigenvectors. Every eigenvector has a corresponding eigenvalue. Ranked by the eigenvalues, the eigenvector of the first principal component of data matrix that possesses the maximum eigenvalue is selected. The FPC curve is obtained by mapping the data matrix into the first principal component space, which is represented by the selected eigenvector that contains maximum energy or information. That is, the FPC curve is a new vector, which dimension is the same as the data collection times, obtained by using the data matrix multiplying the selected eigenvector. In this study, the curve is employed as a quantitative tool to objectively rate the major depressive mood status of subjects.

### Quantitative model construction for objectively rating the mood status

In this study, the RF regression analysis method is selected to execute the quantitative evaluation model construction task. It can generate binary trees which are made of nodes, every node represents a predictor, and the path from root node to leaf node is intuitive for illustrating the relationship between predictors and quantitative outcomes. In order to ensure the model is able to assess the major depressive mood status accurately, the effectiveness of the model should be evaluated. The model training and testing steps are listed as follows:

1. The LOPOCV is used to split the EEG features and the FPC curve into two parts, respectively. That is, we adopt LOPOCV to split the datasets of 9 subjects into training and testing datasets.
2. Model Training. In every validation, a random forest regression model is trained and built based on the EEG features (independent variable) and the FPC curve (dependent variable) of 8 subjects. The EEG features of the rest subject are inputted into model and generate the quantitative outcomes.
3. Correlation analysis is utilized to estimate the performance of the model. The correlation of actual data (the testing data of FPC curve) and the quantitative outcomes is calculated via correlation analysis. The *P* value is obtained by t test to interpret the correlation value that has no significance in a certain probability. The correlation coefficient r is closer to 1, and the p value is less than 0.05; the quantitative outcomes are more capable to accurately rate the major depressive mood status in the experimental period.

### Modeling for knowledge sharing

#### Ontology construction

**Fig. 2:**
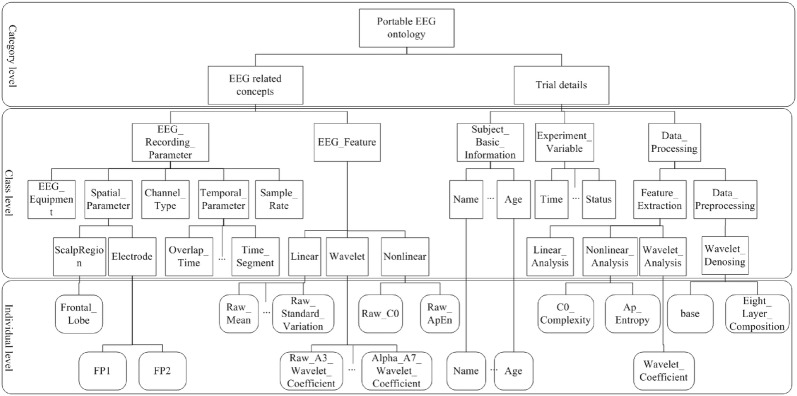
The structure of portable EEG ontology

**Fig. 3:**
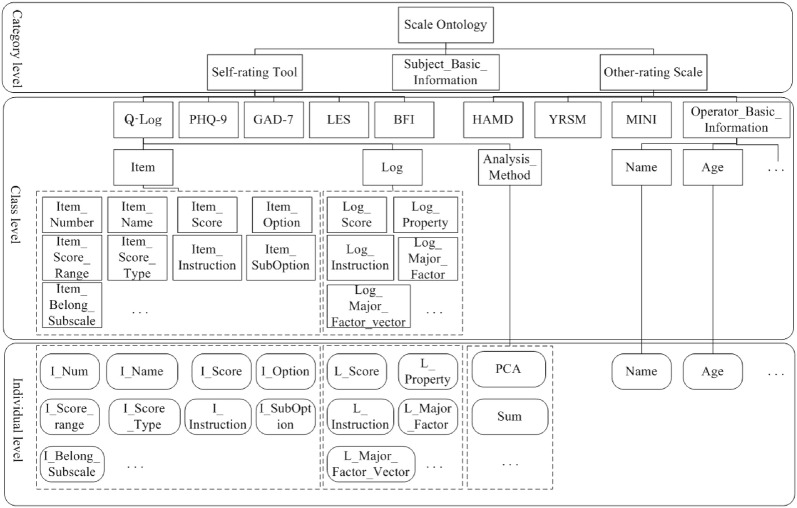
The structure of clinical evaluation tool ontology for depression

Knowledge sharing is defined as a function which is not only able to provide contextual information for the users, but also can offer data annotations for facilitating the users, who are not adept at machine learning technology, to understand the related meaning. Thus, the fundamental work for actualizing the knowledge sharing function is to annotate the contextual information and physiological feature of patients by constructing ontologies. In this case, a portable EEG ontology and a ontology of clinical tool for rating the depressive symptoms are constructed by using Protege 4.3. The structure of the portable EEG and the clinical tool ontology are shown in Figs. 2 and 3, respectively. The ontology structure is depicted from three levels: category level, class level and individual level. The category level defines the most abstract entity concepts, which are also known as the parent classes. The parent classes are subdivided into multiple subclasses in the class level. The individual level describes the instances of the subclasses.

For portable EEG ontology, the related EEG concepts and trial details are concluded in the category level. The subclasses of the related EEG concepts depict the information of EEG recording parameter (equipment, scalp region, sample rate, etc.) and EEG features (linear, wavelet and nonlinear). The subclasses of trial details describe the subject information, experiment variable and data processing. The subject information is recorded to define the subject number, name, sexual, the owner–member relationship between the collected dataset and the subject, etc. The information of experiment variable illustrates the detailed experiment method, such as the data collecting time, the experiment status (resting status or task status), the materials used in the experiment. The data processing information describes the related information of extracted features, such as nonlinear feature extraction (C0 complexity and approximate entropy) and data de-noising method.

For clinical tool ontology, three parent classes are introduced in the category level: self-rating tools, subject basic information and clinician-rating scale. In the class level, the structure of subject basic information is congruent with the one in the portable EEG ontology. The classes of self-rating and clinician-rating scale depict all the scales used in this experiment. For example, the MINI is a short structured clinical interview which enables researchers to make the diagnosis for psychiatric disorders. The HAMD is a common clinical other-rating scale which can evaluate the depression symptom. The Young Mania Rating Scale (YMRS) is also a common used clinical other-rating scale which can evaluate the mania symptom. Since we use the Q-Log to subjectively assess the major depression mood status, the ontology structure of the Q-Log is elaborated. In the class level, the Q-Log class contains three subclasses: item, log and analysis method. The item class defines the specific items in the Q-Log, such as item number, item name, item score, item options. The log class describes the detailed Q-Log information, such as scale score, scale instruction, property, the major emotional factor. The analysis method class depicts the mathematical statistics method adopted for processing the Q-Log data. The individual level instantiates the subclasses defined in the class level.

Besides the ontology structure, the object property is designed to describe the relationship between individuals, and the datatype property is designed to link the individual with a kind of numeric data. Some definitions of the object and datatype property in the portable EEG ontology are illustrated in Tables 1 and 2, respectively.

**Table 1:** Part of the object properties in portable EEG ontology

Object property	Domain	Range
*hasEEGFeature*	*Subject*	${EEG\_Feature}$
*containSubFeature*	*Nonlinear*	${Raw\_C0}$
*onElectrode*	${EEG\_Feature}$	*Electrode*

**Table 2:** Part of the datatype properties in portable EEG ontology

Datatype property	Domain	Range
*hasValue*	${EEG\_Feature}$	*double*
*deviceName*	${EEG\_equipment}$	*string*
*hasName*	*Subject*	*string*

#### Deriving data connotation using Jena inference subsystem

The core idea of using Jena inference subsystem is to facilitate the clinician to derive contextual information and connotation of physiological features. The subsystem is designed to allow a range of inference engines to be plugged into Jena. Such engines are used to derive additional Resource Description Framework (RDF) assertions which are entailed from some base RDF together with any optional ontology information and the axioms and rules. In this study, the provenance information about the extracted features and the relationship between features and quantitative outcomes are described and provided. For actualizing that, three steps are designed:

1. Summarize the rules from the random forest regression model and transform them into Jena format. Because the random forest model is consisted of several decision trees, several rules which number is equivalent with the number of trees can be generated. That is, every decision tree could be transformed into a rule in certain format used in Jena.
2. According to the formatted rules, the Jena inference API is used to derive the notation from the built ontologies. Based on the preceding work about annotating the concepts and properties, the Jena inference API can be used to read these annotations from ontologies. An inference model is created to query the information on the specified meaning of features and the relationships between feature and quantitative outcomes.
3. Organize the annotations and provide them as a feedback to clinician. Combined with the contextual information of subject, we can create a Java API to automatically organize the annotations and provide them to clinicians or other users.

## Results

### Depressive mood status rated by the Q-Log

**Fig. 4:**
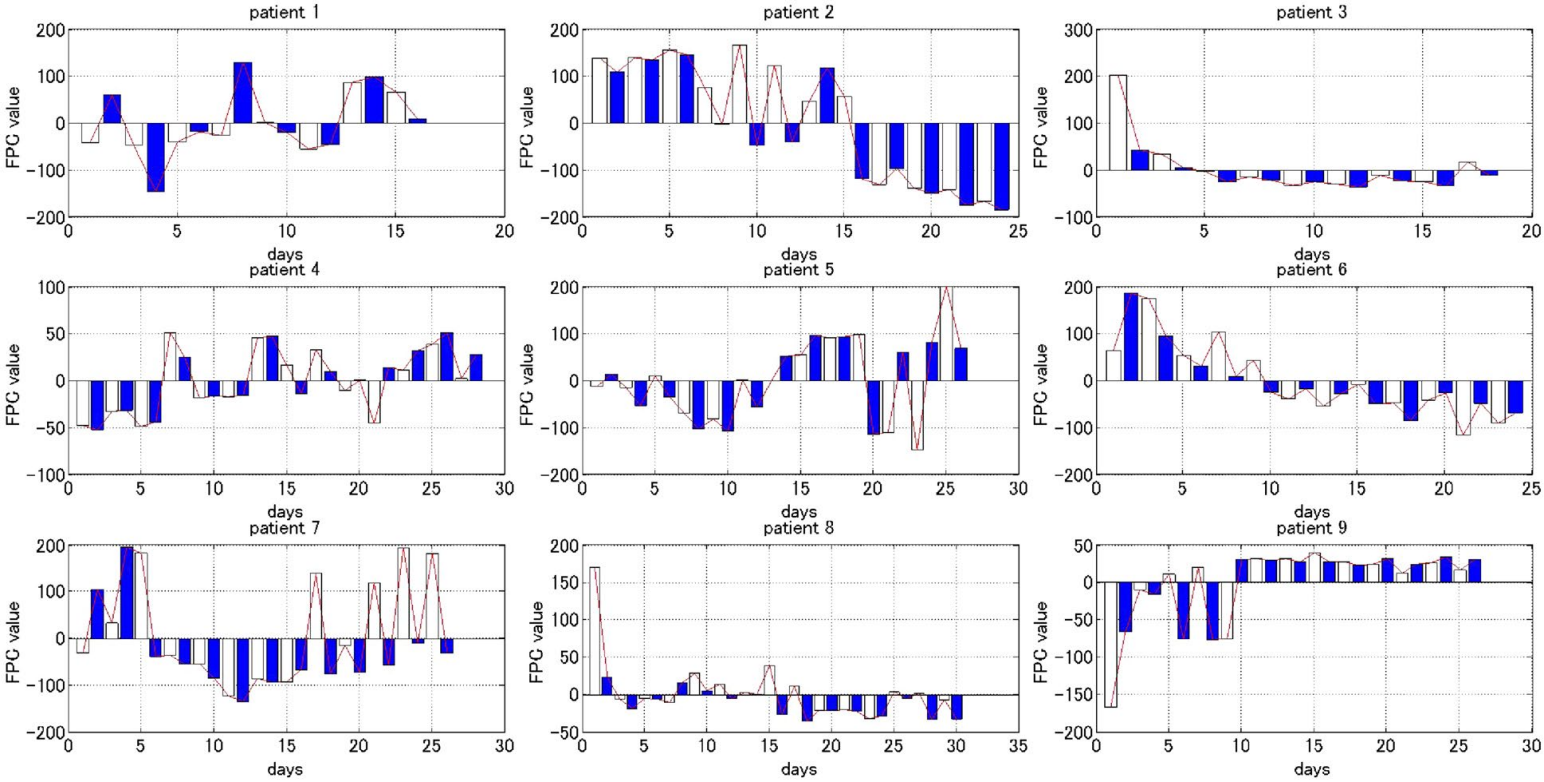
The overall tendency and local variation of the FPC

The FPC curve of the Q-Log is able to tell us the fluctuation of the major depressive mood status self-rated in the experimental period of subjects. Figure 4 shows the overall tendency and local variation of the FPC curve. For each subfigure, the horizontal coordinate indicates the collecting time of the Q-Log, and the vertical coordinate shows the FPC values which implies the quantitative value of depressive mood status rated every time. We can conclude that (1) for patients 2, 3, 6 and 9, the overall tendency of those curves declined, which indicates the depressive symptom is ameliorated after 2 weeks of therapy. In contrast, for patients 1, 4, 5 and 7, the overall tendency of those curves declined in the first week but rebounded in the second week, which means the depressive severity is not remitted after accepting the drug therapy of 2 weeks; (2) bar graph shows the local variation of FPC curve. The scores in the FPC curve have two types: the value of depressive mood status rated in the morning, which is marked by the white bar, and the value of depressive mood status rated in the evening, which is marked by the blue bar. As shown, for the patients whose condition is ameliorated, the value rated in the morning is higher than the value rated in the evening. This rhythm is more obviously observed in the first week. This conclusion is congruous with the clinical experience, which shows the feelings of the depressive in the morning are often worse than the evening. After discussing with clinicians, the evaluation result reflected by FPC curve for each inpatient is congruent with the real condition.

### Quantitative model evaluation

#### Parameter selection for RF

**Fig. 5:**
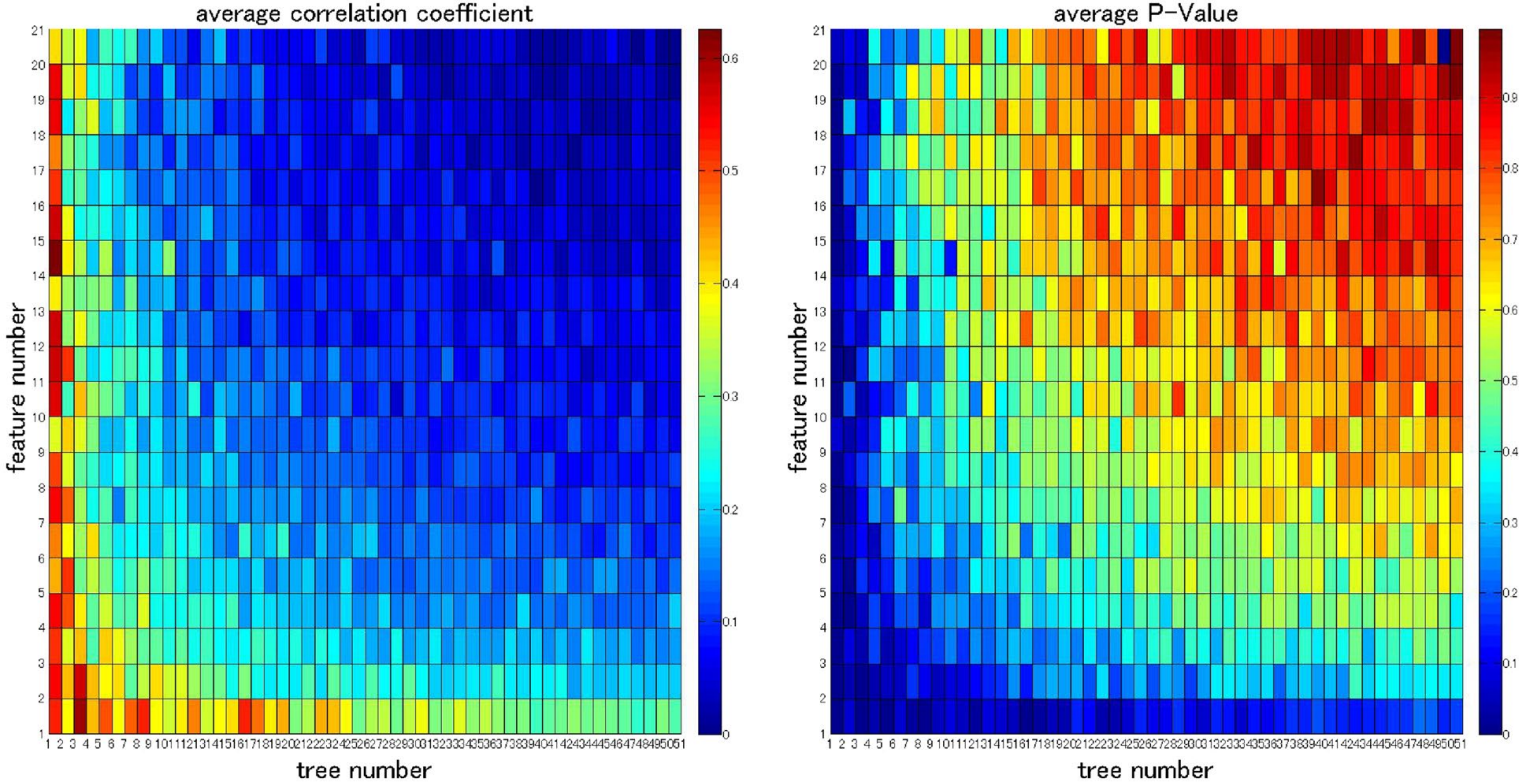
Meshgrid plot of average correlation coefficient and average *P* value

For actualizing a pervasive method for objectively rating depressive mood status as effective as the Q-Log, the RF algorithm is used to construct a regression model to objectively rate the depressive mood status based on the forehead EEG features and the FPC values. The performance of the RF is highly affected by the inputted parameters, especially the number of features (mtry) and the number of trees (ntree) formed a tree and forest, respectively. The grid optimization method is used to select the optimal two parameters. The ntree parameter is set from 1 to 500, and step size is 10. The mtry parameter is set from 1 to 100, and step size is 5. For every LOOPCV, we can obtain a pair of correlation coefficients (CC) and *P* value. In order to find out the parameter pair with optimal CC and *P* value, we average the CCs and *P* values of 9 times and select the pair with maximum CC and minimum *P* value as the optimal parameter pair. Figure 5 shows the meshgrid plot of average correlation coefficient and *P* value when various feature number and tree number are selected. According to Fig. 5, the best CC and *P* value are obtained when ntree equals to 1 and mtry equals to 66. This indicates that one tree with high depth is sufficient to obtain the effective outcomes for quantitatively rating the depressive mood status. In succeeding work, we choose (ntree = 1, mtry = 66) as the optimal parameter pair to train the regression model and obtain quantitative outcomes.

#### Model evaluation using t test

Table 3 shows the results about the effectiveness evaluation of quantitative model. TrS means the sample size of training data, and TeS indicates the sample size of testing data. Because the size of collected samples is different for each patient, the number of TrS and TeS is also different in every LOPOCV. The correlation coefficient and *P* value are the results of correlation analysis obtained by t test. The average CC of 9 inpatients is 0.6556, and the standard deviation is 0.0476. The *P* values are all below 0.01, which implies that there is less than 1% probability happened in chance that the quantitative outcomes for objectively rating the major depressive mood status have a moderately strong uphill relationship with FPC values. This result may provide a promising evidence to demonstrate that the built quantitative model is feasible to objectively rate the depressive mood status as effective as the Q-Log.

**Table 3:** Result about the effectiveness evaluation of quantitative model

Patient	TrS	TeS	CC	*P* value
1	202	16	0.7238	$<0.01$
2	194	24	0.6096	0.0016
3	200	18	0.7146	$<0.01$
4	190	28	0.6259	$<0.01$
5	192	26	0.6102	$<0.01$
6	194	24	0.5969	0.0021
7	192	26	0.6791	$<0.01$
8	188	30	0.6537	$<0.01$
9	192	26	0.6873	$<0.01$

### An exemplary application for knowledge sharing

According to the quantitative model, the trees contained in the model are manually transformed into rules in Jena format, and Jena inference APIs are used to derive feature notations from built ontologies. Because the tree in quantitative model has a high depth, a rule with long length is transformed from the tree. As the length limit, the rule is simplified to give an exemplary illustration. The simplified rule is given and explained in Table 4.

**Table 4:** An exemplary rule extracted from RF model

rule1: (? subject rdf: type base: Subject) (?v1 rdf: type base: original_min_CD6) (?v1 base: hasValue ? value1) greaterThan (?value1, -0.2539) (?v1 base: onElectrode ? point1) (? point1 rdfs:label “FP1”) (? v2 rdf: type base: alpha_psd) (?v2 base: hasValue ? value2) greaterThan (? value2, -0.1422)(?v2 base: onElectrode ? point 1) (? point1 rdfs: label “FP1”)(?v3 rdf: type base: denoised _sum_CD4) (?v3 base: hasValue ? value3) greaterThan (? value3, 0.3396) (?v3 base: onElectrode ? point 1) (? point1 rdfs: label “FP1”)(? RealValue rdf: type base: MoodStatus) (? RealValue base: has MoodStatus Value ? value4) $->$ AssignOne (? value4, ? MoodStatus)	

The words with prefix $'?'$ (such as $'?v1'$, $'?v1'$, $'?value1'$ ) are the variables defined in Jena; $'base:'$ indicates the class name or property name defined in the ontology, such as the $'Subject'$ is the subject class, $'hasValue'$ is one of defined properties. The classes of the EEG feature are also followed by $'base:'$. For example, the feature named $'original\_min\_CD6'$ is the minimum of the sixth layer wavelet coefficient, which indicates the minimum wavelet decomposition coefficient of the theta subband. The ‘denoised_sum_CD4’ means the sum of the fourth layer wavelet coefficient, which means the energy of the beta subband. The Jena functions are also depicted in the rule, such as $'greaterThan'$ and $'AssignOne'$. The general meaning of the rule is that supposing the feature $'original\_min\_CD6'$ , $'alpha\_psd'$ and $'denoised\_sum\_CD4'$ of the Fp1 channel is greater than $-0.2539$, $-0.1422$ and 0.3396, respectively. Then a quantitative value of the major depressive mood status could be outputted by the model and assigned to the variable $'MoodStatus'$.

## Discussion

In this paper, an objective and pervasive method driven by the WaaS architecture is proposed to provide a daily quantitative evaluation for depressive mood status. Nine inpatients are recruited to collect forehead EEG and Q-Log data. The PCA method is used to process the Q-Log data of each patient and obtain the FPC curve, which is able to depict the overall tendency and local variability of depressive mood. The result shows that FPC values of each patient have a dynamic global variation, and the local variation can be observed by comparing the value of morning and evening in a single day. It is worthy to highlight that we summarized the results of each patient from the FPC, and discuss them with clinicians who did not demur our conclusions and support the intensive study in hospital. Actually, the Q-Log belongs to Ecological Momentary Assessments (EMA), which is an electronic diary method. Participants are repeatedly assessed for a certain period of time (usually days to weeks), by administering a single or a set of questionnaires on a relatively high frequency (e.g., daily or multiple times per day) [37–41]. Similarly, the dramatic feature of the Q-Log is that it can be self-rated in a daily granular, which facilitate clinicians to observe the mood status change of inpatients more detail. Markowetz et al. mentioned that the temporal granularity at which traditional methods collect data commonly is too coarse to reveal fine-granular patterns [10]. The Q-Log provides a possible way to execute the identical psychometric test multiple times over the course of a single day and reveal a daily pattern for the depressive mood status.

Other researchers have previously explored the combination of self-rated scale data with sensor data [37, 38, 42–45]. The result demonstrates that the combination could provide new insights into the pervasive and objective depression evaluation processes in daily life. Based on the FPC curve and the features extracted from forehead EEG data, we adopted RF regression algorithm to construct a quantitative model for objectively rating the depressive mood status (independent variable: the EEG features and dependent variable: the FPC curve). The FPC curve is used as a reference standard to evaluate the quantitative model. The LOPOCV method is used to split the dataset into training and testing datasets. For every LOPOCV, the model evaluation is finished by the correlation analysis which is utilized to quantify the correlation of the quantitative outcomes and the FPC curve of the rest subject. The result demonstrates that the quantitative outcomes for objectively rating the major depressive mood status have a moderate uphill relationship with FPC values, which provides a promising evidence to illustrate the built quantitative model that is feasible to objectively rate the major depressive mood status.

For applying the pervasive method in clinical field of depression, an exemplary application named knowledge sharing is actualized. A portable EEG ontology, ontology of clinical tool for rating the depressive symptoms and related object and property are constructed using Protege 4.3. The constructed quantitative model is manually transformed into Jena format. The Jena API is used to derive feature notation from built ontologies. Previous studies always adopt an ontology model to build the terminology of depression and utilize the Bayesian networks or other machine learning methods to infer the probability of depression [46–49]. Our technique route gives a feasible way to build a platform to integrate data from commercially available sensors with clinical psychological data. Combined with the advantages of ontology and quantitative analysis method, researchers could have a new perspective to cope with depression assessment and therapy.

However, there are several limitations in this paper: (1) The EMA assessment is always conducted from a large population sample at one or a few points in time. The Q-Log data of 9 inpatients are absolutely insufficient to make it being applied to the real clinical practice; (2) in every LOPOCV, the constructed pervasive model is different for every inpatient. A uniform model trained based on fixed dataset is more appreciate for objectively rating the major depressive mood status; (3) because our platform is being developed, the graphic application interface is unable to be provided. The exemplary application for knowledge sharing given in the 3.3 section is not intuitive to illustrate the usage of data annotation. Our future work includes the following directions: (1) More subjects should be recruited so as to test the clinical value of Q-Log in the intensive study; (2) corresponding to the Q-Log data, the forehead EEG samples should also be further collected to search effective quantified feature or feature set and build the uniform quantitative model; (3) platform development is necessary for showing the advantage and effectiveness of ontology and quantitative analysis method.

## Conclusions

This study aims at developing a pervasive analysis method following the WaaS architecture for objectively rating the depressive mood status using portable EEG device and self-rating tool. The data analysis result of the self-rating tool named Q-Log demonstrates that the tool provides an effective evaluation performance for the depressive mood status. The regression analysis method based on RF method is used to construct the quantitative model for objectively rating the depressive mood status. The result shows that the model outcomes have a moderate uphill relationship with the FPC values of the Q-Log, which gives an evidence to support the model effectiveness. Furthermore, an exemplary application of knowledge sharing, which is developed by using ontology technology and Jena inference subsystem, is given to illustrate the preliminary work for annotating data and facilitating clinical users to understand the meaning of the quantitative analysis results. This method combining physiological sensor data with psychological self-rating data could provide new insights into the pervasive and objective depression evaluation processes in daily life.
